# Echocardiographic assessment of left ventricular function in ex situ heart perfusion using pump-supported and passive afterload working mode: a pilot study

**DOI:** 10.1186/s44158-021-00018-3

**Published:** 2021-12-02

**Authors:** Arnaud Romeo Mbadjeu Hondjeu, Azad Mashari, Ryan Ramos, Giulia Maria Ruggeri, Bryan Gellner, Roberto Vanin Pinto Ribeiro, Joshua Qua Hiansen, Frank Yu, Liming Xin, Mitchell Brady Adamson, Mitesh Vallabh Badiwala, Massimiliano Meineri

**Affiliations:** 1 Department of Anesthesia and Pain Management, Peter Munk Cardiac Center Toronto General Hospital, University Health Network, Toronto, Canada; 2 Department of Mechanical and Industrial Engineering, University of Toronto, Toronto, Canada; 3 Division of Cardiovascular Surgery, Peter Munk Cardiac Center, Toronto General Hospital, University Health Network, Toronto, Canada; 4 Department of Surgery, Faculty of Medicine, University of Toronto, Toronto, Canada; 5 Department of Anesthesia and Intensive Care, Herzzentrum Leipzig, Strumpell Strasse 39, 04289 Leipzig, Germany

**Keywords:** Echocardiography, ex vivo heart perfusion, heart transplant, Left Ventricle

## Abstract

Ex situ heart perfusion (ESHP) has been developed to decrease cold ischemia time and allow metabolic assessment of donor hearts prior to transplantation. Current clinical ESHP systems preserve the heart in an unloaded condition and only evaluate the cardiac metabolic profile. In this pilot study we performed echocardiographic functional assessment using two alternative systems for left ventricular (LV) loading: pump supported afterload working mode (SAM) and passive afterload working modes (PAM). Six hearts were procured from male Yorkshire pigs. During cold ischemia, hearts were mounted on our custom made ESHP circuit and a 3D-printed enclosure for the performance of echocardiography with a standard TEE probe. Following perfusion with Langherdorf mode of the unloaded heart, the system was switched into different working modes to allow LV loading and functional assessment: pump supported (SAM) and passive (PAM). Echocardiographic assessment of left ventricular function in the donor hearts was performed in vivo and at 1 h of ESHP with SAM, after 4.5 h with PAM and after 5.5 h with SAM. We obtained good quality epicardial echocardiographic images at all time points allowing a comprehensive LV systolic assessment. All indices showed a decrease in LV systolic function throughout the trial with the biggest drop after heart harvesting. We demonstrated the feasibility of echocardiographic functional assessment during ESHP and two different working modes. The expected LV systolic dysfunction consisted of a reduction in EF, FAC, FS, and strain throughout the experiment with the most significant decrease after harvesting.

## Introduction

Heart failure (HF) is one of the leading causes of death worldwide [1]. According to the National Health and Nutrition Examination Survey data from the USA, an estimated 6.2 million adults presented with HF between 2013 and 2016 compared to 5.7 million between 2009 and 2012 [1]. While various treatment strategies have been implemented to improve symptoms and decrease mortality, cardiac transplantation remains the treatment of choice for patients with advanced refractory HF [2].

Over the last two decades, the number of suitable donor hearts has plateaued while the demand for organs continues to increase [3]. This has motivated strategies to expand the donor pool such as the utilization of hearts procured through donation after circulatory death (DCD). The main burden to donor heart harvesting after DCD is the warm ischemia occurring between the withdrawal of life-sustaining treatment and reperfusion or cardioplegia [4]. Although cold ischemic storage is the universally accepted method of DCD heart preservation, it is not ideal. Low levels of anaerobic metabolism continue in the background with subsequent depletion of adenosine triphosphate (ATP) stores and an increase in acidosis [5]. Combined with the warm ischemic insults already impacting DCD donor hearts, this may significantly decrease organ function after transplant [6]. Therefore, it is critical in this scenario to improve myocardial preservation and reperfusion prior to transplantation.

Ex situ heart perfusion (ESHP) has been introduced as a technique with the potential to improve heart transplant outcomes by reducing cold ischemic time and supporting aerobic metabolism, thereby allowing better and longer allograft preservation between retrieval and implant [7, 8]. The Organ Care System (OCS) is currently the only available system for clinical human ESHP. It allows the preservation of a donor heart in Langendorff (LM) or resting mode. LM consists of delivering perfusate in a retrograde fashion from the aortic root to the coronary arteries without loading the left ventricle (LV) and relies solely on metabolic parameters such as lactate extraction to determine if the heart is suitable for transplant [9]. Given the lack of ventricular loading, the OCS is not suitable for functional assessments of myocardium at this time [10]. As a result, several groups are in the process of developing new ESHP systems to assure more physiological allograft perfusion with ventricular loading to allow functional assessment and quantification of cardiac mechanics in order to predict organ suitability for transplant [11, 12].

Heart function is determined by a complex interaction between preload, afterload, heart rate and the inotropic state of the myocardium. Our group has developed and validated a novel modular ESHP system to allow functional donor heart evaluation with biventricular loading (working mode) [13–15]. This novel modular ESHP system can produce physiological hemodynamic characteristics and evaluate contractile parameters in both the left and right ventricles of adult-sized porcine hearts in three different modes: LM, biventricular pump-supported working mode (Bi-SAM) and biventricular passive afterload working mode (Bi-PAM).

LV functional assessment has been traditionally achieved experimentally using transduction catheters to obtain pressure-volume loops, allowing quantification of ventricular elastance during LV loading on ESHP [16]. The predictive value of these measurements for outcomes after transplantation is still unknown [17]. In addition, transduction catheters have several limitations: they are costly, invasive, and can only be placed in isolated hearts. Echocardiography is the gold standard for the perioperative assessment of cardiac function. However, in the setting of ESHP it has only been reported as a marginal component of the overall cardiac evaluation [18]. We developed a custom-made 3D-printed enclosure to support and protect the donor heart during ESHP and permit epicardial imaging using a standard transesophageal echocardiography (TEE) probe [19]. ESHP with controlled loading may allow a standardized and non-invasive assessment of the LV during working mode and may increase the early identification of organ dysfunction prior to transplantation and thereby improve patient outcomes.

Up until now, the validity of using SAM to assess the cardiac function is controversial as the retrograde aortic flow is not physiological and uncontrollable rises in systolic pressure may impact heart function. By allowing a more physiological perfusion of LV, PAM has been proposed as an alternative to SAM that strives to improve the physiological appropriateness of LV afterload during ESHP [14, 15]. A standardized setting is fundamental for a reliable functional assessment of the heart during ESHP and for determining if these hearts are usable for transplantation. The relative feasibility and physiologic significance of functional assessment under the two working modes is not currently known. In this study, we sought to assess the feasibility of performing a reliable and comprehensive functional assessment of LV during ESHP using echocardiography in both afterload working modes.

## Methods

### Animal preparation

This pilot study was conducted on six male Yorkshire pigs (50.2 ± 5.98 kg Caughell Farms, ON, CA). The experimental protocol was approved by our institutional animal care committee and followed the ARRIVE guidelines [20]. Animals were treated as per the “Guide for the Care and Use of Laboratory Animals”. All animals received humane care in compliance with the Principles of Laboratory Animal Care formulated by the National Society for Medical Research and the Guide for the Care and Use of Laboratory Animals prepared by the Institute of Laboratory Animal Resources.

### Anesthesia, monitoring, and baseline measurements

Animals were acclimatized for at least 7 days. The night before experiments, they were fasted. In all animals, premedication was provided with an intramuscular injection of midazolam (0.3 mg/kg), ketamine (20 mg/kg), and atropine (0.04 mg/kg). Anesthesia was induced using inhalational isoflurane (end tidal concentration 1–3%) and maintained on the same concentration through an oral endotracheal tube and mechanical ventilation was established with a tidal volume of 6–8 ml/kg. An arterial line was inserted through the right common carotid artery and a central venous line was introduced into the left jugular vein. A pulmonary artery catheter was inserted via the right jugular vein and directed into position beyond the pulmonary artery bifurcation. Standard monitoring consisted of: EKG, pulse oximetry, end tidal CO_2_ and invasive arterial pressure. After a median sternotomy and pericardiotomy, the heart and great vessels were exposed. An umbilical tape was placed around the inferior vena cava and a pressure-volume conductance catheter (Millar Instruments Inc., Houston, TX, USA) was inserted into the left ventricular apex. Systemic anticoagulation was achieved with an intravenous injection of heparin (30,000 IU).

### Heart donation

Following in vivo baseline echocardiographic evaluation, a purse-string suture was placed in the ascending aorta to allow placement of a cardioplegia cannula. An 18F venous cannula was inserted into the inferior vena cava (IVC) via the right atrium for collection of approximately 1.5 L of whole blood into an auto-transfusion system (Frensenius Kabi C.A.T.S., Terumo, USA) to isolate the red blood cells (RBC). Simultaneously, the aorta was cross-clamped, and donor hearts arrested with 1 L of histidine-ketoglutarate-tryptophan (HTK) solution at 4 °C. The donor heart was weighed, excised, and placed in ice-cold HTK for one hour while being cannulated for the ESHP circuit.

### Ex situ set-up and protocol

The donor heart was then connected to the ESHP system cannulas that were suspended in a 3D-printed custom-made holder. The circuit composed a reservoir (Affinity Fusion®, Medtronic, Minneapolis, MN), an oxygenator (Affinity Fusion®, Medtronic, Minneapolis, MN) and two centrifugal pumps (560A and 540 T, Medtronic, Minneapolis, MN, USA) working in parallel to selectively load the LV and right ventricle (RV), as described above. A heat exchanger (Sarns Dual Heater Cooler Model 11,160) maintained a stabile temperature of 37 °C.

The system was primed with 2000 mL of whole autologous blood from the donor and normal saline to achieve a hematocrit > 25%. Methylprednisolone (500 mg), heparin (10,000 IU), cefazolin (1 g), and magnesium (2 g) were also added to the priming. Calcium chloride 10%, sodium bicarbonate 8.4% and dextrose 50% were added to correct calcium (1.1–1.3 mmol/L), glucose (5–10 mmol/L), and bicarbonate (24–30 mmol/L) concentrations, respectively.

### Perfusion modes

During LM, oxygenated perfusate is pumped retrograde by a centrifugal pump into the aorta at a constant pressure of 50 mmHg that results in aortic valve closure and perfusate flow into the coronary vessels. The perfusate drains into the coronary sinus and through the right ventricle it is ejected back to the reservoir via a cannula into the pulmonary artery [13]. In this mode, the LV is not loaded and cannot therefore be functionally evaluated.

The working mode allows for LV loading and functional assessment. During diastole, SAM enables both antegrade flow to the left atrium and retrograde flow into the aorta. The retrograde flow is provided by a pump allowing coronary perfusion. In systole, the same retrograde flow acts as aortic resistance. However, the LV must overcome the aortic backpressure which can cause an uncontrolled rise in aortic systolic and diastolic pressure. PAM is an alternative to SAM that may simulate systemic vascular resistance more closely by connecting the ascending aorta to a Windkessel-based afterload module. In an electrical system, the Windkessel module comprises a circuit containing lumped elements of resistance, capacitance, and inductance. Here, the governing equations of an electric circuit are applied to a fluid system, where fluid pressure, fluid volume and volumetric flow rate directly parallel voltage, electrical charge, and electrical current, respectively [21]. A physical Windkessel module can possibly provide more realistic and predictable vascular impedances for in vitro flow experiments [22]. It is used for computational fluid dynamics validation and other investigations of the cardiovascular system and medical devices [23]. A Windkessel module describes the hemodynamics of the arterial system in terms of resistance and compliance [24]. Increasing resistance results in an increase in both systolic and diastolic pressure. Increasing compliance results in a decrease in systolic pressure and an increase in diastolic pressure. Through manipulation of resistance and compliance, systolic and diastolic pressures can be varied independently. In PAM, measured in vivo aortic, systolic, and diastolic pressures are targeted while in SAM, the diastolic pressure is maintained at 30 mmHg and the systolic pressure is not controlled.

### Protocol

**Fig. 1:**
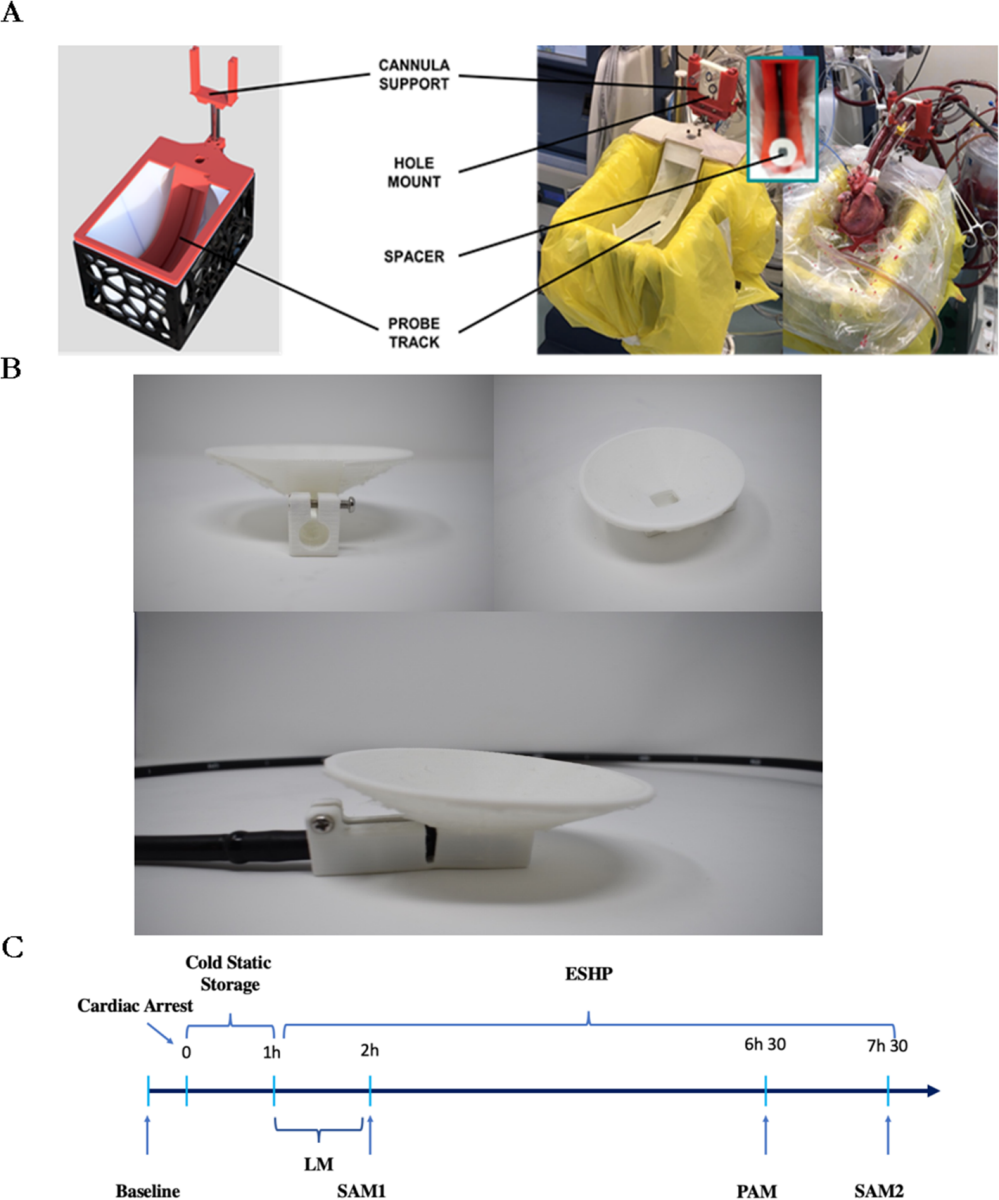
Experimental design showing **A** setup, **B** custom made 3D-printed spacer, and **C** experimental timeline

In this experiment, the donor heart was mounted on the ESHP system following 1 h of cold storage. Anterograde flow through the LA was commenced for de-airing of the left cardiac chambers. Once completed, all hearts were first perfused in LM. Over the course of 30 min, hearts were rewarmed to 37 °C. Hearts that fibrillated were defibrillated as required. Perfusion was kept steady at these settings for another 30 min. The metabolic assessment of the donor heart was performed hourly in LM. For the functional assessment, the hearts were transitioned to SAM as described previously and the LA loaded with an inflow corresponding to a cardiac index of 1.8 L/min/m^2^ based on donor weight. The right atrium was loaded with an inflow such that the sum of the inflow and coronary flow corresponded to a cardiac index of 1.8 L/min/m^2^ based on donor weight. The centrifugal pump and left atrial resistance were adjusted such that diastolic pressure was maintained between 25 and 30 mmHg. Systolic aortic pressure was not controlled. A Windkessel module was added on the aortic line in order to regulate systolic and diastolic aortic pressure. The evaluation process was performed with a standardized systemic resistance of 2 mmHg/cm^3^ and a standardized arterial compliance of 2 cm^3^/mmHg. The LV was assessed in SAM at 1 h (indicated as SAM1), then switched to LM. After 4.5 hours was assessed in PAM and after 1 h of LM (5.5 h of perfusion) the hearts were switched back into SAM2 to perform the final functional assessment. Figure 1A shows the detailed setup.

Continuous infusions of dobutamine (0.05 mcg/min), nitroglycerin (1 mcg/kg/min), and insulin (5 units/h) were maintained throughout the experiment. Fraction of inspired oxygen (FiO2) and gas flow through the oxygenator were titrated to maintain a pH between 7.35 and 7.45, a pO2 between 100 and 300 mmHg, and a pCO2 between 35 and 45 mmHg. Arterial and venous samples were collected at baseline and hourly during ESHP. Electrolyte, lactate and hemoglobin concentrations, pH, pO_2_, pCO_2_, and oxygen saturation (SpO_2_) were measured using a blood gas analyzer (RAPIDPoint® 500 Blood Gas Systems, Siemens). Myocardial lactate metabolism was determined as follows:

Myocardial Lactate Metabolism [mmol/L] = Pulmonary Artery Lactate [mmol/L] − Aortic Lactate [mmol/L]

A positive result indicates production, whereas a negative result indicates extraction. All metabolic parameters were assessed with hearts perfused in LM. The heart was weighed at the beginning and at the end of the perfusion.

### Echocardiographic assessment

A standard TEE probe (TEV5Ms Transducer) and echocardiography system (Acuson SC2000, Siemens, Mountain View, CA) were used to perform a comprehensive epicardial echocardiographic examination. In order to allow for an optimal interface between the donor heart and the probes, a custom-made 3D-printed spacer was applied at the tip of the transducer, filled with gel. The probe was covered by a disposable sterile sheath. Figure 1B illustrates the custom-made 3D-printed spacer and Fig. 1C depicts the experimental timeline. We were able to obtain the following standard echocardiography views: apical 4 chamber (Ap4), apical 2 chamber (Ap2), apical 3 chamber (Ap3), apical 5 chamber (Ap5), LV short axis (LV SAX), and LV long axis (LAX). For the assessment of LV function, we focused on 3 views. The LV short axis view was obtained with the probe positioned behind the LV at 0°. This view is equivalent to TEE trans gastric LV short view. It was followed by the 2-chamber view where the probe was maintained behind the LV at 90°. The 4-chamber view was obtained by advancing the probe to the LV apex and anteflexed while maintaining the angle at 0°. LV function was quantified according to current guidelines [25] using biplane ejection fraction (EF), fractional shorting (FS), fractional area change (FAC), global longitudinal strain (GLS), global circumferential strain (GCS), and radial strain (RAD). Strain was measured offline with Echo Insight software (Epsilon Imaging, trademark, Ann Arbor, MI, USA). Global strain was computed as the average of the strain values of all LV segments. FS, FAC, GCS, and RAD were measured in the LV SAX. The use of TEE probe to perform epicardial echocardiography on an isolated heart during ESHP allows minimal manipulation of the heart, and the echocardiographer to stay away from the set-up, therefore consenting illimited direct manipulation of the heart from the experimental team

### Statistical analysis

We reported the trend in measured parameters at the different time points using the mean and standard deviation. All statistical analyses and graphics were generated using MATLAB (MATLAB and Statistics Toolbox Release 2018a, The MathWorks, Inc., Natick, MA, USA).

## Results

### Echocardiographic assessment of LV

**Fig. 2:**
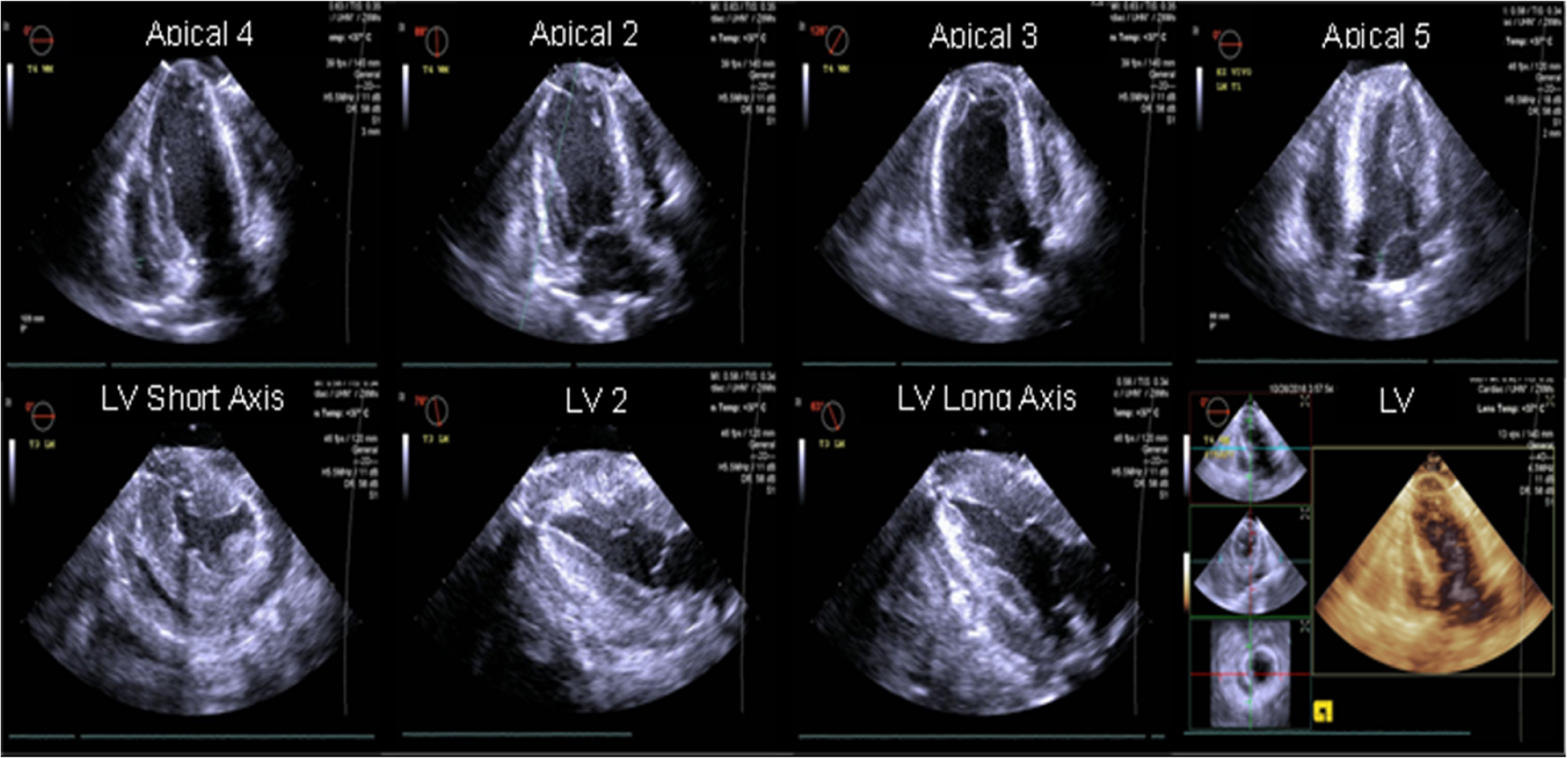
Representative echocardiographic images obtain with our 3D-printed set-up in working mode during ESHP. ESHP, ex situ heart perfusion; LV, left ventricular

**Fig. 3:**
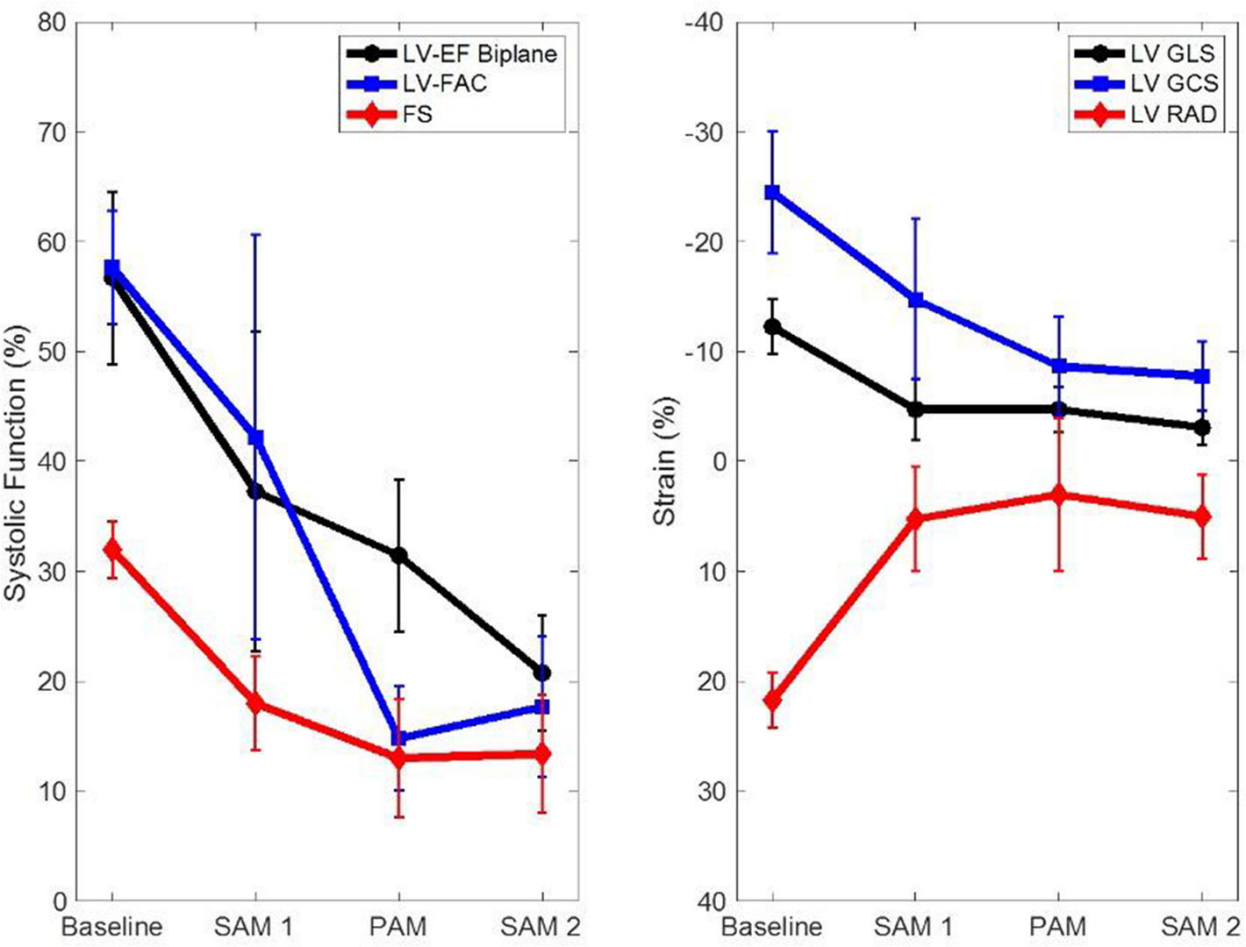
Left ventricular functional assessment showing **A** EF, FAC, and FS; **B** strain. Error bars show the standard deviation. SAM, pump-supported working mode; PAM, passive afterload working mode. LV, left ventricular; EF, ejection fraction; FAC, fractional area change; GLS, global longitudinal strain; GCS, global circumferential strain; RAD, radial strain; FS, fractional shortening

In all six pigs included in the study, we successfully completed all assessments and successfully loaded the ventricles transitioning from unloaded non-working (LM) perfusion mode to two different working modes by loading the left and the right atrium. At all timepoints, we were able to obtain good quality epicardial echocardiographic images that allowed a complete systolic assessment of LV (Fig. 2). Overall, all indices showed a decrease in LV systolic function throughout the trial (Fig. 3; Table 1).

**Table 1:** Comparison of echocardiographic parameters measured during baseline, SAM1, PAM, and SAM2

Parameter	Baseline Mean ± SD	SAM1 Mean ± SD	PAM Mean ± SD	SAM2 Mean ± SD
LV-EF biplane	56.71 ± 7.86	37.28 ± 14.56	31.44 ± 6.90	20.76 ± 5.23
LV-FAC	57.64 ± 5.16	42.19 ± 18.38	14.78 ± 4.73	17.66 ± 6.44
FS	31.98 ± 2.57	17.97 ± 4.29	12.99 ± 5.38	13.37 ± 5.34
LV GLS	− 12.26 ± 2.50	− 4.72 ± 2.81	− 4.71 ± 2.07	− 3.08 ± 1.60
LV GCS	− 24.50 ± 5.57	− 14.75 ± 7.32	− 8.67 ± 4.51	− 7.75 ± 3.20
LV RAD	− 21.75 ± 2.50	− 5.25 ± 4.78	− 3.00 ± 7.00	− 5.00 ± 3.83

Values displayed are mean ± standard deviation. *SAM* pump-supported working mode, *PAM* passive afterload working mode, *LV* left ventricular, *EF* ejection fraction, *FAC* fractional area change, *GLS* global longitudinal strain, *GCS* global circumferential strain, *RAD* radial strain, *FS* fractional shortening

The LVEF was 56.71% ± 7.86, 37.28% ± 14.56, 31.44% ± 6.90, and 20.76% ± 5.23 at baseline, SAM1, PAM, and SAM2, respectively. The LVFAC was 57.64% ± 5.16, 42.19% ± 18.38, 14.78% ± 4.73, and 17.66% ± 6.44 at baseline, SAM1, PAM and SAM2, respectively. The LVFS was 31.98% ± 2.57, 17.97% ± 4.29, 12.99% ± 5.38 and 13.37% ± 5.34 at baseline, SAM1, PAM and SAM2, respectively.

GLS was − 12.26% ± 2.50, − 4.72% ± 2.81, − 4.71% ± 2.07, − 3.08% ± 1.60 at baseline, SAM1, PAM, and SAM2, respectively. The GCS was − 24.50% ± 5.57, − 14.75% ± 7.32, − 8.67% ± 4.51, − 7.75% ± 3.20 at baseline, SAM1, PAM, and SAM2, respectively. The RAD was − 21.75% ± 2.50, − 5.25% ± 4.78, − 3.00% ± 7.00, − 5.00% ± 3.83 at baseline, SAM1, PAM and SAM2, respectively.

### Metabolic and macroscopic evaluation during ESHP

**Fig. 4:**
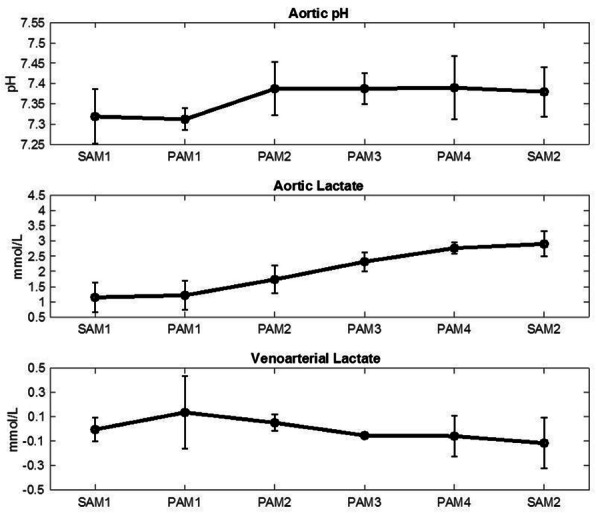
Metabolic parameters measured. **A** Aortic pH, **B** Aortic lactate, **C** Venoarterial lactate. Error bars show the standard deviation. SAM, pump-supported working mode; PAM, passive afterload working mode

The arterial pH was measured during the seven hours of reperfusion. With the aggressive management of sodium bicarbonate and CO_2_, pH remained relatively stable during this time period (Fig. 4a). As seen in Fig. 4b, lactate concentration increased continuously during SAM and PAM. Lactate concentration (in mmol/L) was 1.15 ± 0.49, 2.77 ± 0.18, and 2.90 ± 0.41 at SAM1, PAM, and SAM2, respectively. Arterial and venous lactate concentration measurements were used to calculate the lactate extraction (Δlactate) at the different time points. The Δlactate (in mmol/L) between the venous and arterial perfusate samples was − 0.01 ± 0.1, − 0.06 ± 0.17, − 0.12 ± 0.21 at SAM1, PAM, and SAM2, respectively (Fig. 4c). As seen in Table 2, myocardial weight increased during the experiment.

**Table 2:** Macroscopic evaluation of the donor heart over the course of the experiment

Pig weight (kg) Mean ± SD	Baseline heart weight (g) Mean ±SD	Post-ESHP heart weight (g) Mean ± SD	Heart weight gain (g) Mean ± SD	Heart weight gain (%) Mean ± SD
50.2 ± 4.0	248.5 ± 27.2	333.8 ± 21.0	85.25 ± 23.6	35.13 ± 10.1

Values displayed are mean ± standard deviation. *ESHP* ex situ heart perfusion

## Discussion

In each of the six experiments, the system demonstrated stability across the trial. Metabolic parameters were maintained within a physiological range. The hearts demonstrated predominantly an aerobic metabolism throughout the experiments as demonstrated by the neutral lactate metabolism and the stable lactate levels across the perfusion period.

The capability to mitigate the effect of warm ischemia during the withdrawal of life-support, the necessity to preserve the heart during transportation from the donor to the recipient, and the need to assess the viability of the heart are key for the success of DCD transplantation. Currently, the average ischemic time is around 4–6 h [26]. However, a successful transplantation of a heart following preservation time of 10 hours was reported by Stamp et al. [27]. With increasing ischemic time, donor hearts are at an increased risk of primary graft failure (PGF). In our study, the time to load the LV and perform the echocardiographic assessment of the LV was approximately 30–45 min. The decision of final assessment at 4 h and 30 min for the PAM and 5 h 30 min for the SAM2 is within the time frame for average ischemia in previously published papers.

To allow donor heart exchange in the largest area possible from Toronto, we decided for the final assessment after 5 h 30 min of ESHP. This is the approximate time needed to transport donor hearts across the most populous geographic area in Canada.

We were able to complete a comprehensive echocardiography assessment with SAM and PAM without interfering with the operators and manipulating the heart. This proved the effectiveness of our custom-made setup for echocardiographic assessment during ESHP. Looking at the trend in echocardiographic parameters throughout the trials, the expected LV systolic dysfunction consisted of a reduction in EF, FAC, FS, and RAD as well as in GLS and GCS throughout the experiment. However, the bigger drop was from baseline to ESHP and was partially due to cold ischemia. During the 5.5 hours of ESHP we detected by echocardiography a slow decrease in LV systolic function which is consistent with the metabolic trends and while excludes heart reconditioning, given the difference of only one hour between PAM and SAM2 towards the end of our experiment we would assume that the heart condition would be similar. When comparing the LV parameters at PAM and SAM2 we noticed a larger difference in load dependent parameters such as LVEF when compared to strain. This is likely a confirmation that the two working modes while both allowing functional heart assessment may not be comparable due to different setup and loading conditions.

Reliable, easy to use, and reproducible methods are required to evaluate the myocardial function during ESHP prior to transplantation. Numerous approaches allow the assessment of organ viability during ESHP, including biomarkers of tissue injury (i.e., lactate and troponin I), metabolic measurements (i.e., myocardial oxygen consumption), and hemodynamic and contractility parameters (i.e., pressure-volume loops and echocardiography) [28]. In clinical practice, the heart is preserved in a unloaded mode which does not allow for evaluation of contractile function. The assessment of the allograft with the OCS utilizes therefore only lactate levels and veno-arterial lactate extraction as markers of heart viability and suitability for transplantation [17]. This is sustained by the work of Hamed and colleagues, who found that a serum lactate level above 4.96 mmol/L was a strong predictor of graft dysfunction at 30 days [29] and has been used to identify suitable DCD hearts for clinical transplantation [5]. However, lactate concentration only demonstrated weak to moderate correlations and correlated with fewer outcomes compared with hemodynamic parameters. Both Dornbierer [28] and White [10] have reported similar findings of the limited applicability of metabolic measurements. Biomarkers of myocardial damage like troponin I and creatine kinase-MB have proven to be of limited value in predicting organ viability due to their natural elevation with the warm ischemia and preservation insult.

The evaluation of heart structure and function prior to the cardiac transplantation is therefore auspicable to better identify suitable organs. Previous studies have suggested the advantage of contractility measurements over metabolic parameters during ESHP, but these studies were focused on the use of conductance catheters, which provide a broad range of functional parameters and is the traditional gold-standard when researching myocardial performance [12, 14, 23]. However, these are invasive and difficult to utilize, thereby decreasing result reproducibility. Ideally, methods to evaluate suitable donor hearts should be non-invasive, easy to use, and quick to perform. Two-dimensional echocardiography has also been randomly used to obtain qualitative assessments of myocardial contraction [18, 30]; however, a standardized approach to echocardiography in the ex situ perfused heart has not been developed.

Our study demonstrates the feasibility of a complete non-invasive quantitative echocardiographic assessment of LV systolic function during ESHP by transitioning from LM to two different working modes. It is an important step in facilitating a standardized non-invasive functional assessment of the heart during ESHP to predict the suitability for transplantation. It also provides some quantitative measurements that may establish some references to set a benchmark for future research.

## Strengths and limitations

This was the first study to use a non-invasive tool such as echocardiography to comprehensively quantify LV systolic performance during ex situ perfusion in two different working modes in a large animal model. Previous studies have assessed LV function during ESHP using conductance catheters and limited their investigations to SAM. Our experimental model closely mimics the clinical scenario of standard ESHP and assessment, ensuring more readily translatable findings.

Nevertheless, this work has a few limitations. First, our sample was very small. As such, conclusions regarding the use of echocardiographic assessment of LV performance during SAM and PAM should be considered as hypothesis generating work. Second, the echocardiographic measures in vivo are not entirely comparable to those during ESHP given the different setup and loading conditions. Third, although we tried to provide precise loading and inotropic conditions during the functional assessment, we did not closely control heart rate which may have caused increased variability in some of the parameters evaluated. Fourth, for a comparison between SAM and PAM, the assessment of myocardial function should be done in a closer interval of time and randomizing the technique utilized first. Different degrees of LV dysfunction should also be included. Finally, the RV function, which plays a key role in post-transplant heart failure, was not evaluated due to technical challenges in providing controlled RV afterload during ESHP.

## Conclusions

We successfully demonstrated the feasibility of echocardiographic comprehensive functional assessment of LV function in an isolated heart during ESHP using two different working modes. The expected LV systolic dysfunction in the context of heart harvesting consisted of a reduction in EF, FAC, FS, and strain throughout the experiment. SAM and the PAM provide different LV loading conditions which is partially demonstrated by the echocardiographic quantification, and therefore may not entirely be fully interchangeable for functional LV assessment. Our work sets the basis for the integration of echocardiographic imaging into an ESHP clinical platform for a non-invasive functional heart assessment.
